# F11R Expression upon Hypoxia Is Regulated by RNA Editing

**DOI:** 10.1371/journal.pone.0077702

**Published:** 2013-10-16

**Authors:** Michal Ben-Zvi, Ninette Amariglio, Gideon Paret, Yael Nevo-Caspi

**Affiliations:** 1 Department of Pediatric Critical Care Medicine, Safra Children's Hospital, Sheba Medical Center, Tel Hashomer, Israel; 2 Sackler Medical School, Tel-Aviv University, Tel-Aviv, Israel; 3 Sheba Cancer Research Center, Sheba Medical Center, Tel Hashomer, Israel; 4 Mina and Everard Goodman Faculty of Life Sciences, Bar Ilan University, Ramat Gan, Israel; NIGMS, NIH, United States of America

## Abstract

F11R is a cell adhesion molecule found on the surface of human platelets. It plays a role in platelet aggregation, cell migration and cell proliferation. F11R is subjected to RNA editing, a post-transcriptional modification which affects RNA structure, stability, localization, translation and splicing. RNA editing in the 3'UTR of F11R and RNA levels are increased upon hypoxia. We therefore set to examine if RNA editing plays a role in the increase of F11R RNA seen upon hypoxic conditions. We show that ADAR1, but not ADAR2, takes part in the editing of F11R however editing alone is not sufficient for obtaining an elevation in RNA levels. In addition we show that hyper-edited mature mRNAs are retained in the nucleus and are associated with p54^nrb^. We therefore conclude that hypoxia-induced edited RNAs of F11R are preferentially stabilized and accumulate in the nucleus preventing their export to the cytoplasm for translation. This mechanism may be used by additional proteins in the cell as part of the cell's effort to reduce metabolism upon hypoxic stress.

## Introduction

The complexity of higher organisms is achieved by a variety of post-transcriptional and post-translational mechanisms that enhance gene and protein diversity through the generation of alternative products from a single gene and by their effects on RNA and protein processing. RNA editing is one of the post-transcriptional mechanisms that introduces changes in RNA sequences allowing organisms to produce many more gene products and functions than predicted based on the number of genes within their genome[1]. RNA editing is an essential process for adequate development and is particularly widespread in mammals [2,3]. Of the various types of RNA editing, the adenosine-to-inosine (A-to-I) base modification is the most widespread in higher eukaryotes affecting gene expression at several levels by targeting different types of transcripts [4]. There have been reports indicating that the RNA editing levels are modulated by environmental signals [5]. However, very little is known about the molecular pathways that lead to changes in the activity or specificity of the RNA machinery. 

The site-selective modification of adenosines to inosines in RNA molecules is mediated by a family of enzymes termed ADAR (adenosine deaminase acting on RNA). In humans two ADARs (ADAR1 and ADAR2) are responsible for all currently known A-to-I editing activity and they modify RNAs with distinct but overlapping specificities [4]. ADAR1 encodes two isoforms, p110 and p150, created by transcription from different promoters followed by alternative splicing. ADAR1-p150 is transcribed from an interferon-inducible promoter and encodes a 150-kDa protein located mainly in the cytoplasm [6,7]. The shorter constitutively-expressed ADAR1-p110 isoform is found predominantly in the nucleus [8]. Mammalian ADAR2 is constitutively expressed and most abundant in the brain but can be found in many other tissues. ADAR2 is mainly localized in the nucleus. The extent of editing at a particular site may vary during development or may show cell- or tissue-specificity. The main substrate of the ADAR enzymes is double-stranded RNA (dsRNA) formed primarily by self annealing of complementary regions within a single transcript. It is therefore not surprising that the vast majority of the predicted A-to-I editing sites were found within *Alu* elements, non-coding short interspersed elements (SINEs) about 280bp long, which account for more than 10% of the human genome [9]. *Alu* subfamilies share relatively high homology, which renders them as ideal templates for RNA editing as oppositely oriented Alu elements can form dsRNA structures [10]. The function of editing in repeated sequences is not clear. However, since such sequences are often located within transcripts that are processed to protein-coding mRNA, the editing-induced alteration of the RNA structure, stability or localization, may impact protein expression. Indeed, in some cases a silencing effect is exerted through the presence of the edited repeat elements [11]. A nuclear complex containg the p54^nrb^ protein that binds inosine-containing RNAs has been shown to cause the retention of some highly edited RNAs within the nucleus, thereby preventing their export and translation [11,12]. In the case of the mouse cationic amino acid transporter (Cat2) gene, nuclear-retained transcripts became mobilized for export and translation following cellular stress through cleavage of the inosine-containing 3'UTR from the rest of the mRNA [13]. In addition, editing can destroy or create RNA splice sites or modulate alternative splicing patterns [4,14,15].

It has been previously shown that the F11 receptor (F11R) gene is subjected to A-to-I RNA editing in an *Alu* sequence embedded in the 3'UTR of the gene [16,17]. F11R, also known as JAM-A (Junctional adhesion molecule-A), is a cell adhesion molecule (CAM), member of the immunoglobulin superfamily found on the surface of human platelets and determined to play a role in platelet aggregation, secretion, adhesion and spreading [18]. F11R is also present at tight junctions of endothelial cells (EC) where it plays a role in cell-cell adhesion and cell morphology and migration [19]. Park and colleagues found that in hypoxic carcinoma cells that exhibited enhanced angiogenic and metastatic potential, F11R was overexpressed [20]. Gene expression analysis of hypoxic primary human astrocytes revealed high levels of F11R [21]. In breast cancer F11R overexpression has been linked with reduced survival [22]. We have previously found that a human lymphoblastoid (LB) cell line exposed to Deferoaxamine (DFO) which mimics hypoxia, shows increased F11R gene expression. In addition we have shown that upon DFO treatment A-to-I RNA editing levels, occurring in the 3'UTR of the gene, are elevated [16].

 In the current study we asked if A-to-I RNA editing plays a role in the hypoxia-induced expression of F11R. To address this question we silenced or overexpressed ADAR1 or ADAR2 and examined their effect on F11R expression. In addition we inhibited RNA synthesis in the cell in order to understand if increased F11R expression is due to the synthesis of new RNA molecules. We have found that ADAR1 plays a role in the editing of the *Alu* element embedded in the 3'UTR of F11R. In addition we found that hypoxic conditions and editing of F11R are required for the elevated RNA levels of the gene. Upon hypoxia highly edited F11R transcripts were retained in the nucleus associated with p54^nrb^. Our results suggest a mechanism for controlling F11R expression upon hypoxic conditions.

## Results

The F11R (JAM-A) gene undergoes A-to-I RNA editing in an *Alu* element embedded in the 3'UTR of the gene. We have previously shown that upon treatment of a lymphoblastoid (LB) cell line with DFO, RNA editing in F11R is increased. Concomitant to the increased editing, we observed an increase in the mRNA levels of the gene [16]. 

**Figure 1:**
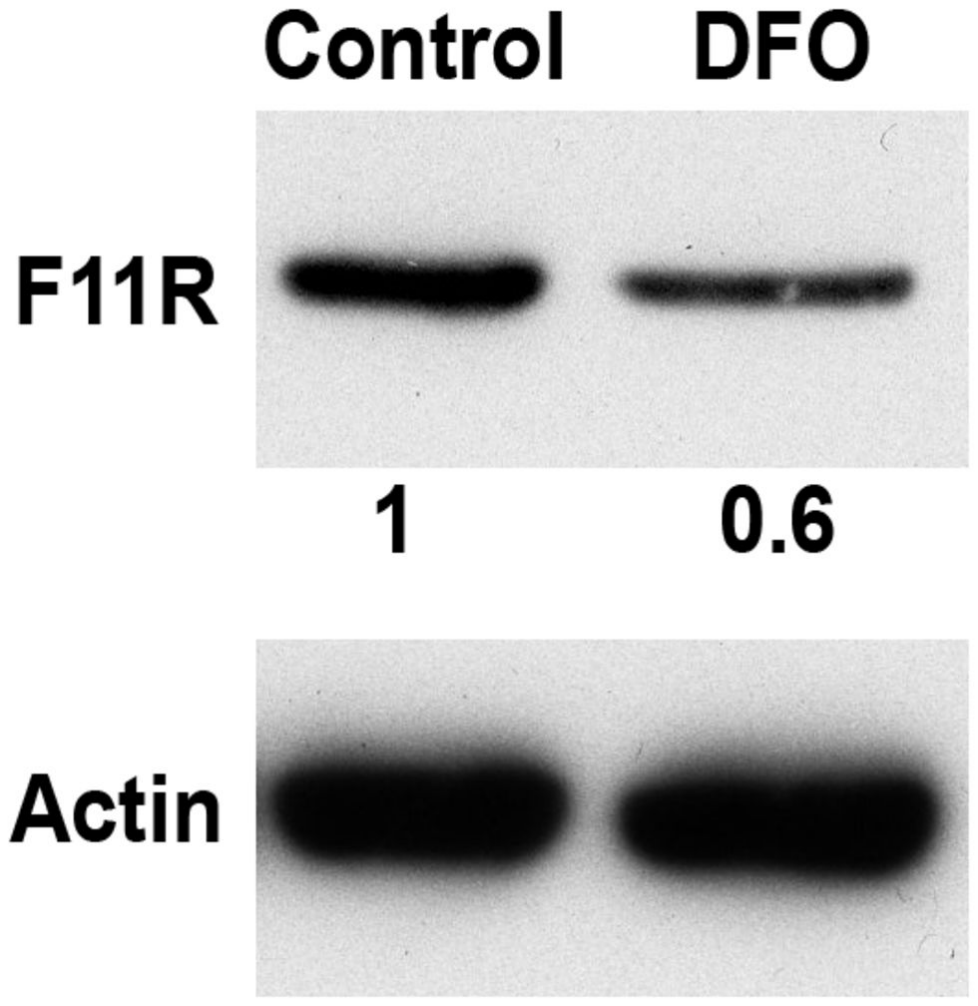
Protein expression of F11R upon hypoxia. Western blot analysis was performed on protein extracts from LB cells 24h following DFO treatment or without treatment. Numbers under the F11R panel represent the relative quantification of the amount of protein. Actin was used as a loading control.

A Western blot of protein extracts from LB cells revealed that, in contrast to the increase in F11R RNA obtained following treatment with DFO, F11R protein levels in treated cells were slightly reduced (Figure 1). 

### Silencing of ADAR1

**Figure 2:**
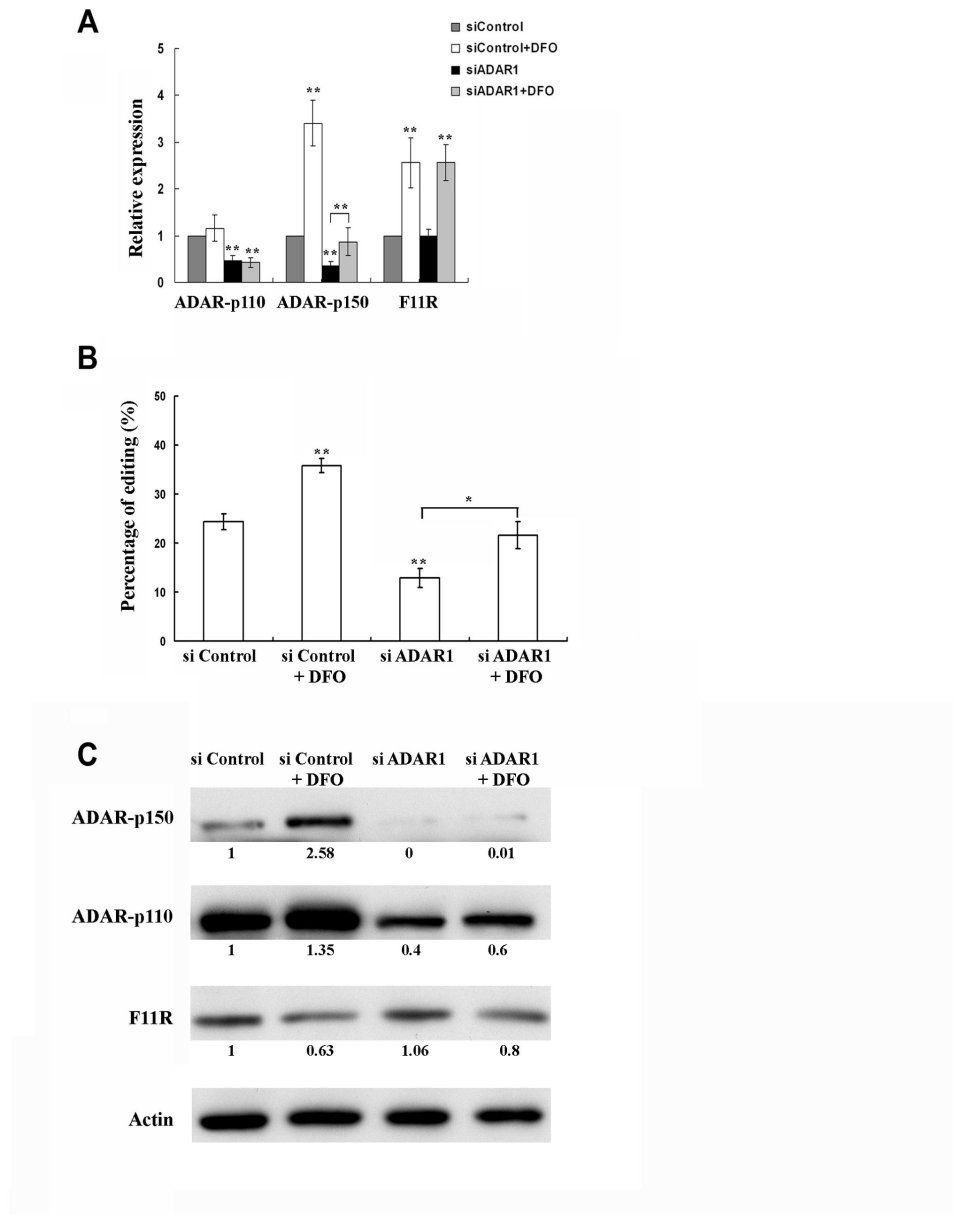
Knock-down of ADAR1. LB cells were transfected with si-ADAR1 or with a control si molecule (siControl). Cells were either treated with DFO 16h post-transfection or untreated. RNA and proteins of all cells were extracted 40h post-transfection (24h post-DFO treatment). Error bars indicate standard deviation (±SD,**p≤ 0.01). (A) Relative quantification of mRNA. mRNA was quantified relatively to the siControl sample which was set as 1. Silenced cells showed low expression of both ADAR1 subunits both in normoxia and upon hypoxia. No effect of the silencing was seen on F11R mRNA which was increased upon hypoxia similarily to the control-transfected cells. (B) Percentage of average RNA editing in F11R upon ADAR1 silencing with or without DFO treatment. ADAR1-silenced cells showed a reduction in editing which was elevated upon DFO treatment however not to the levels seen in the control-transfected cells. p values refer to the difference from the siControl sample unless specified otherwise. (C) Western blot analysis. Numbers in between rows show the relative quantification of the amount of protein set at 1 in the siControl sample. Expression of both ADAR1 subunits was decreased upon silencing. F11R expression was not affected by ADAR1 silencing but was decreased upon DFO treatment. Actin was used as a loading control.

In order to investigate a possible involvement of RNA editing in the regulation of F11R expression upon hypoxia, we silenced ADAR1 in the LB cells using a specific siRNA targeted towards a common region in the transcripts of both ADAR1 subunits: p110 and p150. A decrease of 60-80% in the mRNA and protein levels of both ADAR1 subunits was obtained between 16-48h post transfection. Therefore cells were treated with DFO 16h post transfection and collected 24h post DFO treatment. ADAR1-silenced cells treated with DFO did not show a change in the RNA level of ADAR1-p110 whereas ADAR1*-*p150 RNA was increased 2.5-fold (Figure 2A). ADAR1 silencing resulted in lower editing levels in F11R (Figure 2B). Treatment with DFO, caused an increase in editing however editing levels remained lower than in the DFO-treated control-transfected cells (Figure 2B). ADAR1 silencing did not alter the elevated levels of F11R mRNA seen upon hypoxia nor did it affect the amount of mRNA in normoxia (Figure 2A). These results imply that despite the involvement of ADAR1 in the editing of the 3'UTR of F11R, ADAR1-dependent RNA editing does not play an essential role in the increase of F11R mRNA levels seen upon DFO treatment. Alternatively, since editing was not completely abolished, and was further increased upon DFO treatment, it is possible that the lower amount of editing still occurring in the cell was sufficient to cause an elevation in RNA levels of F11R upon hypoxia. Western blot analysis of the ADAR1-silenced cells revealed that the amount of ADAR1 proteins was indeed reduced and the amount of F11R protein remained similar to the amount seen in the control cells. Treatment with DFO slightly reduced the amount of F11R (Figure 2C).

### Increasing ADAR1 levels

**Figure 3:**
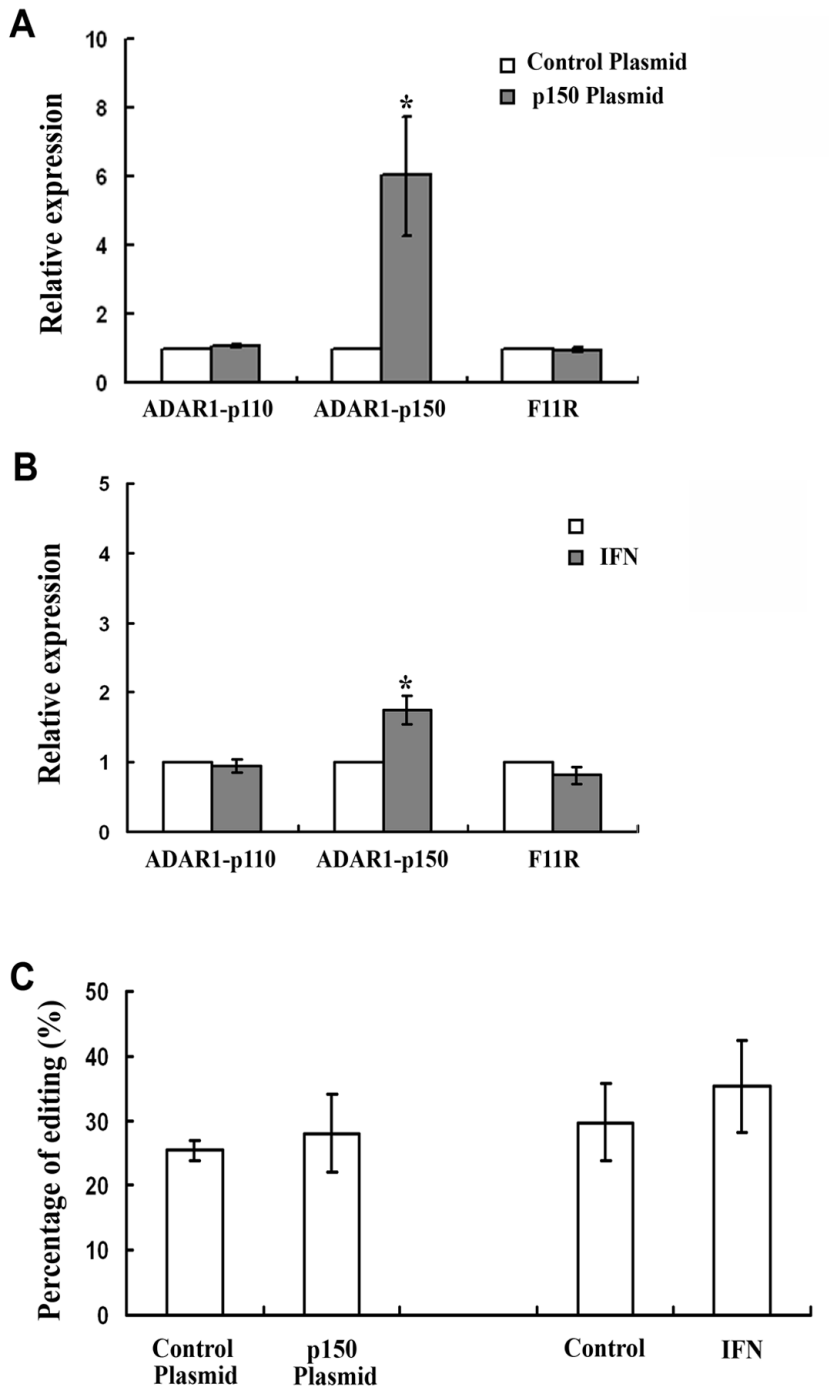
Overexpression of ADAR1-p150 and IFN treatment-α. (A) Relative quantification of mRNA extracted from control cells (transfected with a control plasmid) and cells transfected with a plasmid overexpressing ADAR*-*p150. mRNA was quantified relatively to the control-transfected sample which was set as 1. Error bars indicate standard deviation (±SD,*p≤ 0.05). ADAR1-p150 levels were increased upon transfection however no increase in F11R expression was obtained upon p150 overexpression. (B) Relative quantification of mRNA extracted from control cells and cells treated with IFN-α. mRNA was quantified relatively to the control sample which was set as 1. ADAR1-p150 was increased upon treatment however no increase in F11R expression was obtained upon treatment. (C) Percentage of average RNA editing in F11R upon transfection with a p150-overexpressing plasmid. and upon IFN-α treatment. No significant changes were obtained in RNA editing in F11R.

Since we have shown that upon hypoxia, the amount of the p150 subunit of ADAR1 is increased [16], we examined if other means of increasing the expression of this subunit would result in a similar outcome regarding F11R (e.g. increase in editing and RNA expression). To that extent, LB cells were transfected with a plasmid overexpressing the p150 subunit. The amount of ADAR1*-*p150 RNA increased 4-fold following transfection (Figure 3A), editing levels in F11R did not change (Figure 3C) and the amount of F11R mRNA remained unchanged (Figure 3A). The ADAR1*-*p150 promoter is known to be inducible by IFN-α [7]. LB cells treated with IFN-α exhibited a 1.8-fold increase in ADAR1*-*p150 RNA (Figure 3B). Editing in F11R did not change significantly (Figure 3C) and mRNA levels of the gene remained unchanged (Figure 3B). Our observations suggest that the increase in ADAR1*-*p150 resulting from overexpression or IFN- α treatment is not sufficient in order to increase F11R editing and mRNA levels of the gene. 

### Silencing of ADAR2

**Figure 4:**
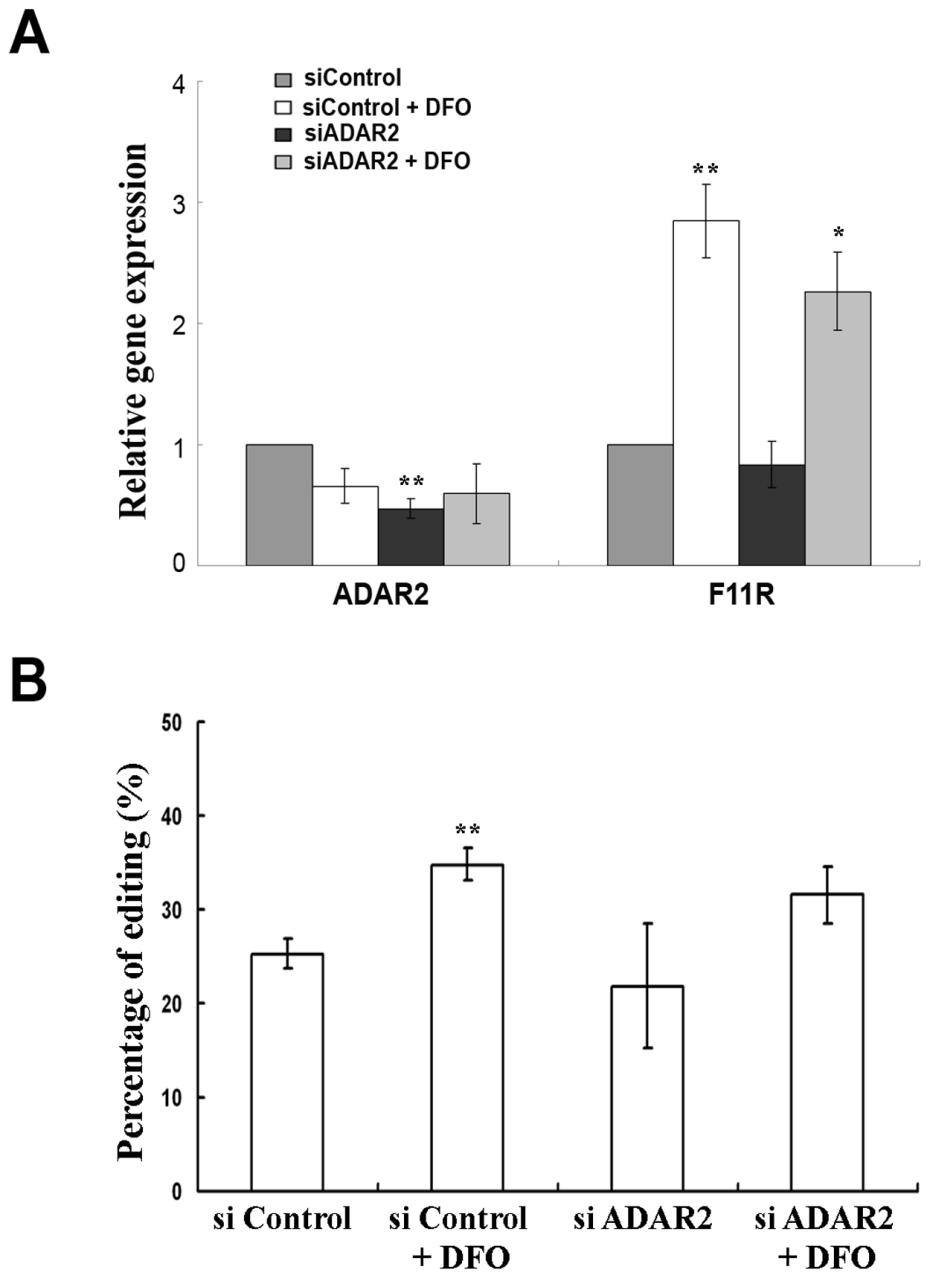
Knock-down of ADAR2. LB cells were transfected with si-ADAR2 or with a control si molecule (siControl). Cells were either treated with DFO 16h post-transfection or untreated. RNA and proteins of all cells were extracted 40h post-transfection (24h post-DFO treatment). (A) Relative quantification of mRNA. mRNA was quantified relatively to the siControl sample which was set as 1. Error bars indicate standard deviation (±SD, *p≤ 0.05, **p≤ 0.01) Silenced cells showed low expression of ADAR2. No effect of the silencing was seen on F11R mRNA which was induced upon hypoxia similarily to the control-transfected cells. (B) Percentage of average RNA editing in F11R upon ADAR2 silencing with or without DFO treatment. ADAR2-silenced cells did not show a change in editing. Editing in the control-transfected cellswas induced upon DFO treatment.

ADAR2 is an additional enzyme known to execute RNA editing. We therefore examined the involvement of ADAR2 in the RNA editing of F11R mRNA. Silencing of ADAR2 in the LB cells caused a reduction of 50% in the RNA of ADAR2. DFO treatment of the ADAR2-silenced cells did not cause a change in ADAR2 mRNA (Figure 4A). Editing in F11R in the ADAR2-knocked-down cells remained unchanged (Figure 4B). DFO treatment of these cells did not cause a change in F11R editing (Figure 4B). The mRNA expression of F11R was not affected by the silencing of ADAR2 upon normoxia or hypoxia (Figure 4A). These results imply that ADAR2 does not play a major role in the editing of F11R sites and is not involved in the mRNA increase of F11R upon DFO treatment. 

### Editing in pre- versus mature mRNA

**Figure 5:**
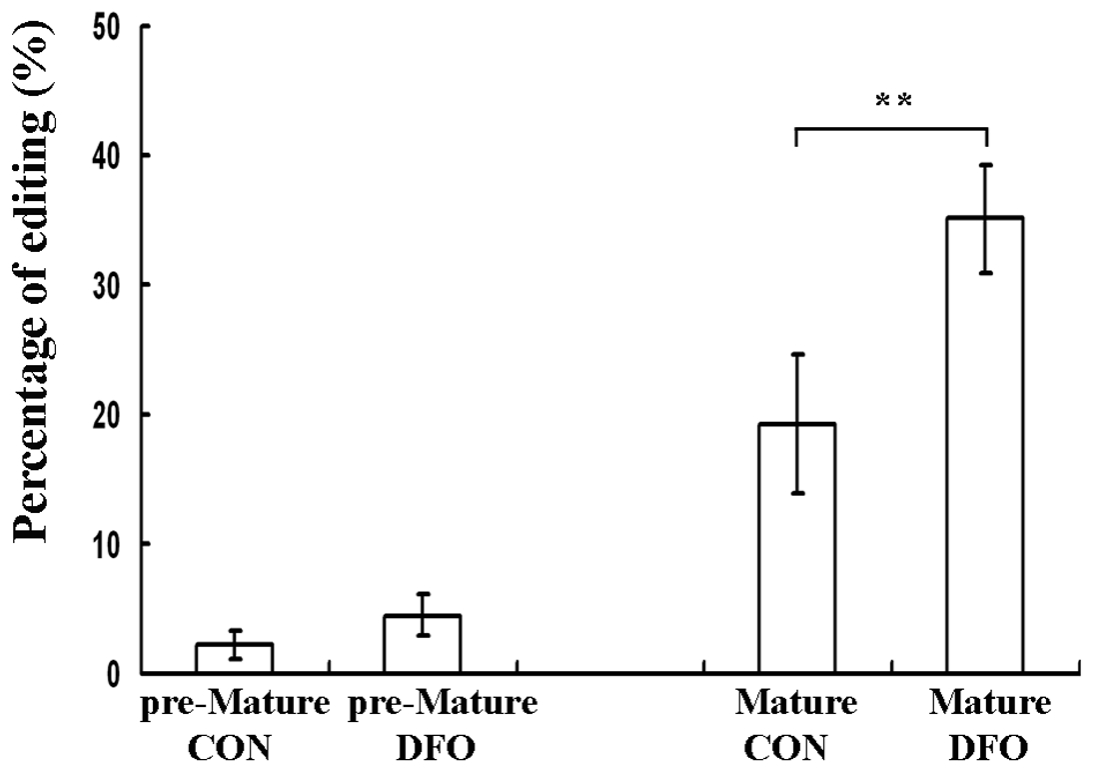
Percentage of average RNA editing in pre- and mature- F11R mRNA . Pre- or mature- F11R mRNA from control and DFO-treated cells was amplified using specific primers. Significant average RNA editing was seen only in mature F11R mRNA which was further increased upon DFO treatment. Error bars indicate standard deviation (±SD, **p≤ 0.01) .

In order to characterize the edited F11R transcripts we amplified the edited F11R region with two sets of primers: the first amplifies the region of interest only from mature mRNA and the second amplifies the edited region only from pre-mRNA molecules. Only the mature F11R mRNA molecules exhibited significant editing which was further increased upon DFO treatment (Figure 5). Since RNA editing is known to occur prior to splicing [15] we conclude that edited F11R mRNA is indeed preferentially spliced. 

### Inhibiting transcription

**Figure 6:**
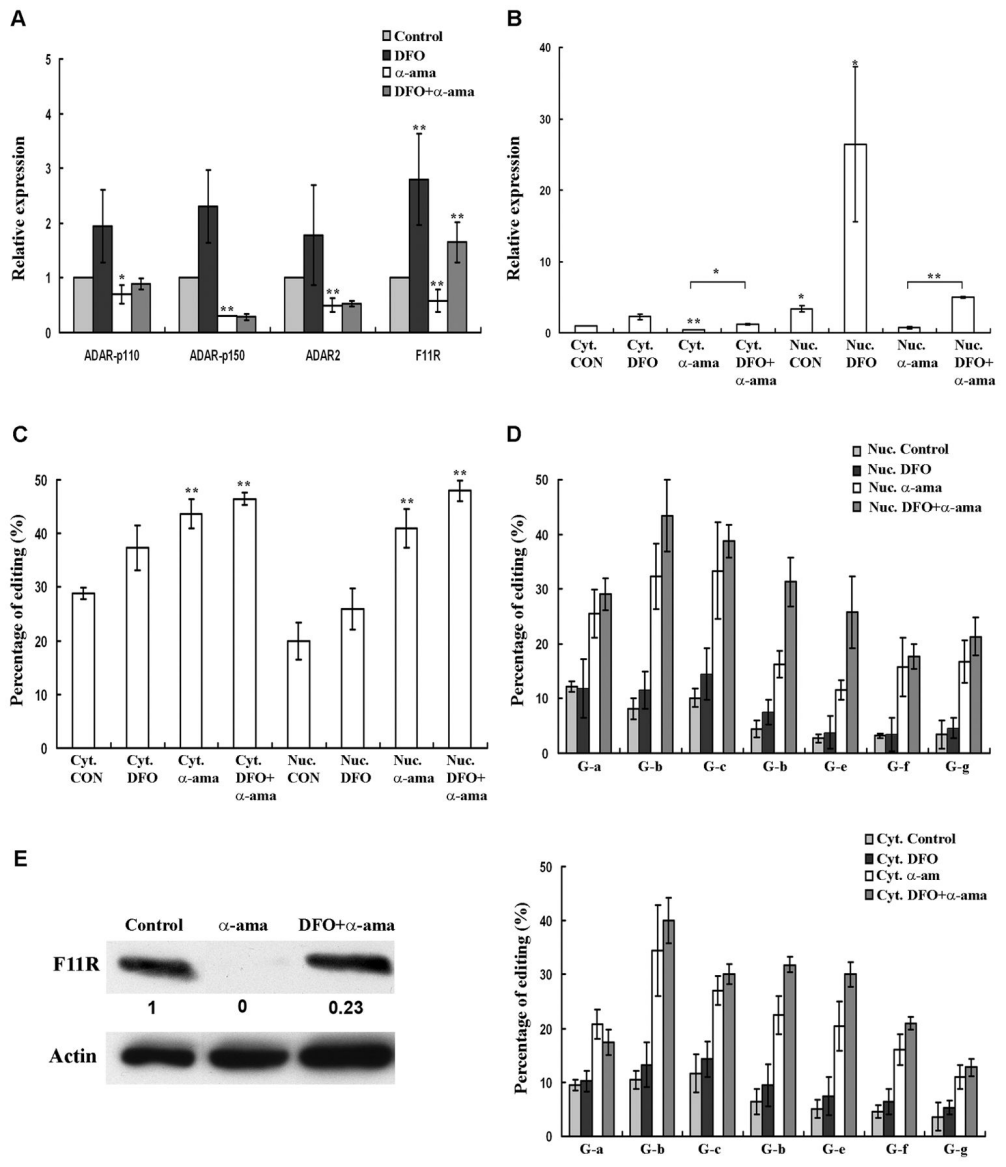
Treatment with α–amanitin. LB cells were treated for 24h with α–amanitin with or without DFO treatment. (A) Relative quantification of mRNA. mRNA was quantified relatively to the non-treated sample which was set as 1. Error bars indicate standard deviation (±SD, *p≤ 0.05, **p≤ 0.01). ADARs and F11R expression was reduced upon α–amanitin treatment. Treatment with both α–amanitin and DFO showed an increase only in F11R mRNA when compared to the amount obtained with α–amanitin treatment only. (B) Relative quantification of mRNA following cell fractionation. mRNA was quantified relatively to the cytoplasmic control sample which was set as 1. Error bars indicate standard deviation (±SD, *p≤ 0.05, **p≤ 0.01). F11R mRNA was higher in the nuclear fractions. α–amanitin treatment reduced the amount of F11R transcripts and the addition of DFO induced them. (C) Percentage of average RNA editing in F11R. Cells treated with α–amanitin showed high levels of RNA editing in F11R. RNA extracted from the cytoplasm showed higher levels of editing when compared to those obtained in the RNA extracted from the nucleus. Error bars indicate standard deviation (±SD, **p≤ 0.01) (D) Percentage of RNA editing in F11R at additional sites. Treatment with α–amanitin revealed significant editing at seven additional sites. Upper panel: editing at these sites in the nuclear extracts following treatment. Lower panel: editing at these sites in the cytoplasmic extracts. Error bars indicate standard deviation (±SD). All the results obtained for the α–amanitin and the α-amanitin+DFO treatments have a p≤ 0.01. (E) Western blot analysis. Numbers in between rows show the relative quantification of the amount of protein set at 1 in the non-treated sample. No F11R protein was seen in the α–amanitin-treated cells. Low F11R expression was seen upon α-amanitin + DFO treatment. Actin was used as a loading control.

Elevated mRNA levels of F11R upon DFO treatment could be the result of *de novo* transcribed molecules or due to the stabilization and accumulation of existing edited transcripts in the nucleus. In order to distinguish between these two possibilities, LB cells were treated with α-amanitin an inhibitor of RNA polII [23]. Following treatment, the amount of the F11R transcripts decreased due to the ongoing degradation of RNAs in the cell and to the inhibition of transcription of new molecules (Figure 6A). The amount of the ADARs transcripts was reduced in a similar fashion. Treating the cells with both α-amanitin and DFO caused a 3-fold increase in F11R RNA (Figure 6A, treatment with α-amanitin and DFO vs. α-amanitin alone) but not in the RNA of ADARs. These results indicate that the DFO-dependent increase in F11R RNA is the result of the stabilization of existing F11R transcripts and not due to newly transcribed molecules. In order to further understand the mechanism contributing to the DFO-dependent stabilization of F11R mRNA, we set to determine the localization of the F11R molecules. To that extent, LB cells were fractionated into nuclear and cytosolic fractions and RNA was extracted from each compartment separately. Upon DFO treatment the great majority of F11R transcripts were found in the nucleus (Figure 6B) suggesting that hypoxia triggers their retention. This observation agrees with the decrease in F11R protein obtained upon DFO treatment (Figure 1). Moreover, the addition of DFO to the α–amanitin treatment caused a 7-fold increase in the number of F11R transcripts in the nucleus, similar to the 7.5-fold increase obtained upon DFO treatment alone, suggesting again that this increase is not the result of newly transcribed molecules. The amount of F11R transcripts in the cytoplasm increased only 2-fold upon DFO treatment reinforcing our observation that a minority of the molecules exits in the cytoplasm upon hypoxia. Editing analysis of the F11R transcripts that remained in the cell following treatment with α–amanitin revealed that the transcripts were highly edited (Figure 6C). In addition to the edited sites that we characterized in the previous experiments we observed significant editing at seven additional sites (labeled a-g, see materials and methods) upon α–amanitin treatment (Figure 6D, upper and lower panels). Hyperedited molecules were detected both in cytoplasm and in the nucleus. We note that although the amount of the cytoplasmic molecules was smaller than their amount in the nuclear fraction (Figure 6B), cytoplasmic transcripts were slightly more extensively edited than those found in the nucleus. This result may indicate that the amount of editing of the transcripts may define the fate of F11R molecules: up to a certain amount of editing they are retained in the nucleus whereas more extensively edited molecules are released to the cytoplasm. Hyperediting of the F11R molecules could be the result of an increase in editing or due to the preferential degradation of poorly edited molecules. Since α–amanitin treatment reduced the RNA of the ADAR enzymes (Figure 6A), we assume that it is likely that the fraction of the edited F11R mRNAs grew due to the degradation of the non- or poorly-edited molecules. Such degradation would leave behind RNA molecules which are significantly edited resulting in our analysis as a higher percentage of editing. It is under such conditions that newly edited sites, previously masked by the non-edited molecules, could appear. These results suggest that highly edited F11R RNAs are more stable than poorly edited transcripts which are preferentially degraded. Western blot analysis did not detect any protein upon α-amanitin treatment (Figure 6E) in accordance with the small number of F11R transcripts in the cytoplasm. However in cells treated with both α-amanitin and DFO the protein could be detected probably due to the slight increase in cytoplasmic F11R RNA.

### RNA immunoprecipitation with p54^nrb^

**Figure 7:**
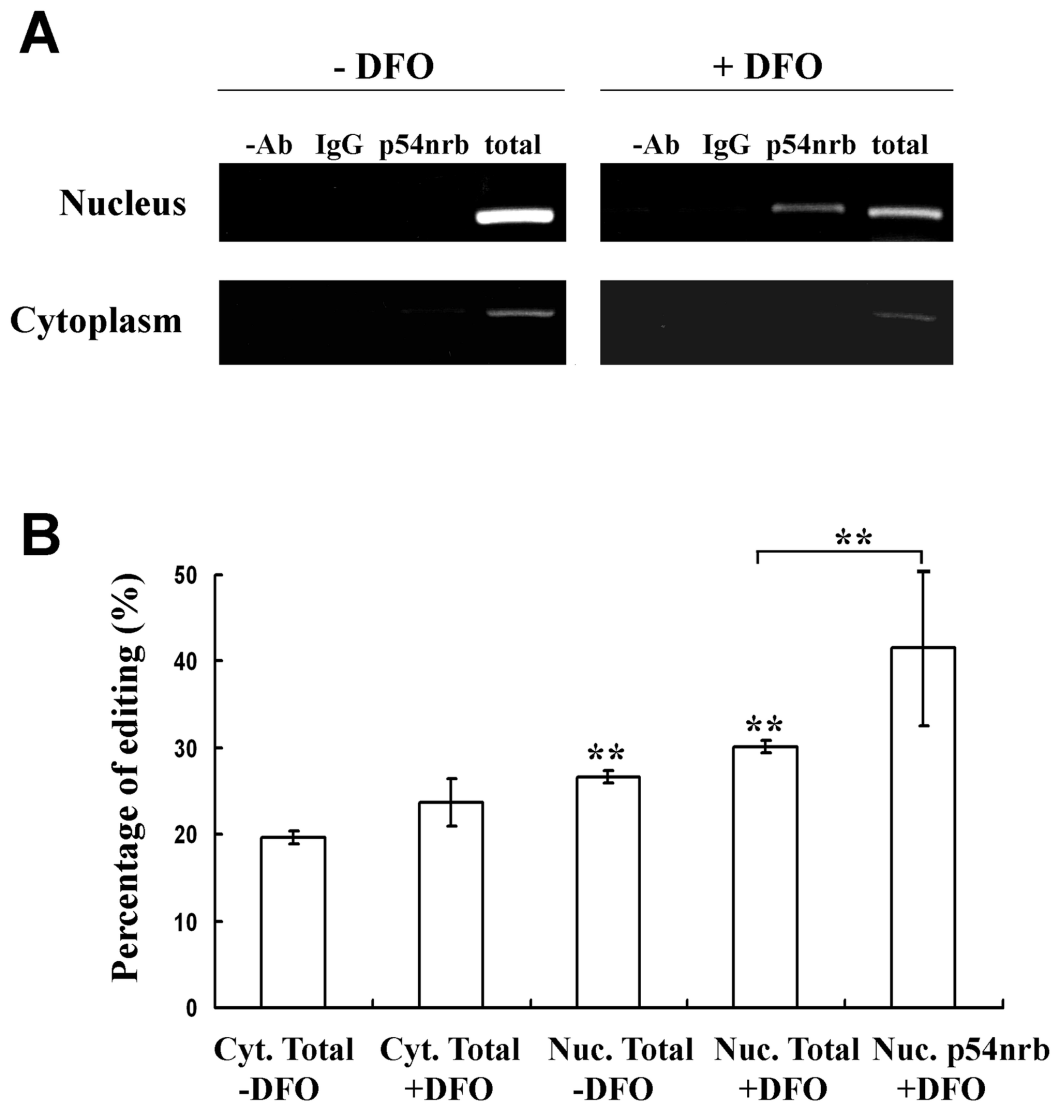
RNA immunoprecipitation with an anti-p54^nrb^ antibody. (A) PCR amplification with F11R-specific primers on RNA extracted from a RIP experiment conducted with an anti-p54^nrb^ antibody on fractionated control cells and fractionated cells treated with DFO. PCR amplification was obtained in the RNA extracted from p54^nrb^-precipitated RNA from nuclear extracts of DFO-treated cells. As a positive control PCR was performed on total RNA (B) Percentage of RNA average editing of F11R RNA that was bound to p54^nrb^. High levels of editing were obtained in the RNA that was extracted from the p54^nrb^-bound molecules.

It has been shown that extensively edited RNAs are retained in the nucleus via an interaction with the p54^nrb^ protein. RNA immunoprecipitation assays with an anti-p54^nrb^ antibody followed by cell fractionation were performed on LB cells with or without DFO treatment. cDNA synthesized from the RNA extracted from such experiments was amplified with F11R-specific primers. A specific band appeared, in addition to the positive controls (total RNA samples), only in reactions conducted on RNA extracted from the nuclei of DFO-treated cells precipitated with a p54^nrb^-specific antibody implying that upon hypoxic conditions F11R RNA binds the p54^nrb^ protein in the nucleus enabling the stabilization of these molecules (Figure 7A). Editing levels of the p54^nrb^-bound RNA molecules was high implying that hyper-edited F11R molecules are preferentially bound to p54^nrb^ (Figure 7B).

## Discussion

F11R is expressed at tight junctions of endothelial and epithelial cells as well as on a variety of hematopoietic cells. Many studies published in recent years have documented the connections between F11R, and its rodent homolog JAM-A, and inflammation, angiogenesis and ischemia/reperfusion. At the cellular level, F11R has been linked to platelet adhesion, cell migration and cell proliferation [24,25]. The RNA levels of F11R have been shown by several investigators (including ourselves) to be increased following hypoxia however the molecular mechanisms leading to this increase and its implications have not been studied [16,20,21]. In this study, we have found that although hypoxic conditions in LB cells cause an increase in RNA levels, protein levels of F11R are not accordingly elevated. This regulation is achieved by the hypoxia-dependent association of extensively A-to-I RNA edited F11R molecules to the p54^nrb^ protein in the nucleus which prevents on one hand their degradation and on the other inhibits their transfer to the ribosomes for translation. 

We show that ADAR1, but not ADAR2, participates in the editing of F11R both upon normoxia and hypoxia. Silencing of the ADAR1 enzymes caused a reduction in editing levels upon normoxia. It is worth noting that in normoxia, although editing was decreased upon silencing of ADAR1, F11R RNA and protein levels were not affected. Upon DFO treatment, editing levels were elevated despite the silencing, albeit they did not reach the editing levels seen in the control experiments. Such editing could be performed by the non-silenced ADAR (ADAR1 or 2, depending on which of them was silenced) or by the small amount of ADAR that remained in the cell despite the silencing (indeed, protein analysis showed that even upon silencing of ADAR1, DFO treatment caused an increase in ADAR1-p150). However, the lower editing levels in the 3'UTR of F11R did not prevent the hypoxia-dependent increase in F11R RNA implying that an additional hypoxia-dependent factor is required for the elevated levels of RNA. Accordingly we have found that overexpressing ADAR1-p150 by means other than hypoxia caused a slight increase in the editing of F11R with no concomitant change in it's RNA levels corroborating our conclusion that hypoxia is required for the elevated levels of F11R RNA. It is worth noting that upon normoxia, silencing of either ADARs and the low editing levels that followed did not affect the amount of F11R RNA implying that these are not a requirement for F11R RNA expression upon normoxia. In addition we show that transcription *de novo* is not essential for the increase in F11R RNA seen upon hypoxia. 

**Figure 8:**
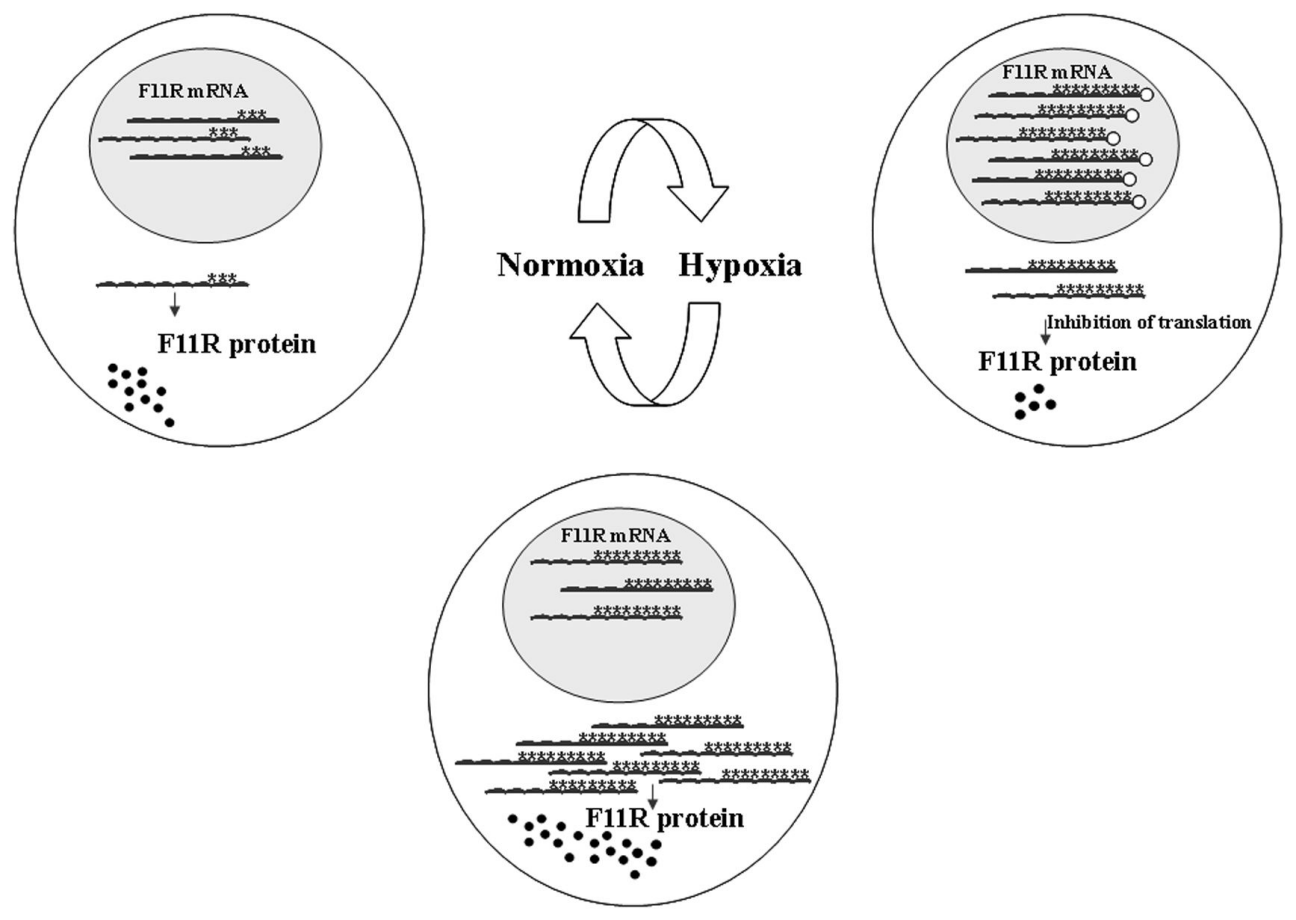
A proposed model for the regulation of F11R expression following hypoxic stress. Upon normoxia, F11R mRNA is transcribed and a fraction of the molecules are exported to the cytoplasm for translation. Upon hypoxia, editing of F11R is increased. The majority of the hyperedited molecules binds p54^nrb^ and is retained in the nucleus in large amounts. Translation of the small number of edited transcripts which escape nuclear retention, is attenuated by a yet to be determined mechanism. We propose that when oxygen levels return to normal, a large amount of F11R hyperedited molecules disengage from p54^nrb^ and thus are released to the cytoplasm for translation enabling protein levels to return to normal very quickly.

So what is the mechanism by which hypoxic conditions cause the increase in F11R transcripts? We have shown that hyper-edited mature mRNA molecules, which are more prevalent upon hypoxia, accumulate in the nucleus and are associated with p54^nrb^ (Figure 8). This result is in accordance with previous studies that have shown that the inosine-specific RNA binding protein p54^nrb^ anchors hyper-edited RNAs to the nuclear matrix [12]. Further studies by Prasanth et al. (2005) have shown that in mice the inosine-containing CTN-RNA is retained in the nucleus with a protein complex including p54^nrb^. Their study, however, shows that following cellular stress, the edited 3'UTR is cleaved and the coding RNA is then released to the cytoplasm and translated to produce the mCAT protein. In our study we show that for F11R, hypoxic stress followed by an increase in editing, serves as a trigger for the association of the edited molecules with p54^nrb^ causing their retention in the nucleus thus controlling export to the cytoplasm and preventing RNA translation. However, while a fraction of the edited molecules binds the p54^nrb^ protein we were still able to detect a small number of molecules which were exported to the cytoplasm in their full length following DFO treatment. This study is in accordance with previous studies showing that edited molecules are exported to the cytoplasm. Work performed in *C. elegans* has shown that mRNAs containing edited structured 3'UTRs can be associated with polysomes despite being edited [26]. Our results show, however, that in our system, although a fraction of the hyperedited molecules reached the cytoplasm upon hypoxia, some mechanism, that is yet to be determined, prevents their translation.

Cells that are no longer under hypoxic conditions undergo a process of deadaptation. It has been shown that rats exhibit regression of cerebral capillary density upon normoxic recovery from chronic hypoxia by a process that involves the activation of programmed cell death and the upregulation of several proteins [27]. A regulated system, such as the one we suggest for F11R, could be implemented by proteins which are required immediately upon hypoxia relief. Such a system would then enable the accumulation of mature mRNAs in the nucleus which can be ready for immediate translation when stress conditions have been resolved.

In conclusion, we suggest that A-to-I RNA editing plays a role in controlling F11R expression upon hypoxic conditions. Hypoxia causes an increase in A-to-I RNA editing in the 3'UTR of the RNA and is the trigger for the association of the edited RNAs to p54^nrb^. In this manner the cell creates a reservoir of F11R mRNA molecules which can be immediately exported, upon need, to the ribosomes for translation without having to waste time on transcription. This mechanism is probably one of many used by the cell in order to reduce metabolism and save energy upon hypoxia.

## Materials and Methods

### Cell line

The cell line used in this work is an Epstein-Barr virus (EBV)-transformed lymphoblastoid (LB) cell line originating from the B cells of a healthy male donor. Ethics statement: The Institutional Review Board (Sheba Medical Center, Tel Hashomer) approved human involvement and cell line creation in this study. Written consent to participate in this study was obtained from donor.

Cells were cultured in RPMI supplemented with penicillin-streptomycin and 10% fetal bovine serum.

### Drug treatments

To mimic hypoxia, cells were treated with 1000μM Deferoxamine (Sigma-Aldrich). 

To inhibit RNA polymerase II transcription cells were treated with 10μg/ml α-amanitin (Sigma-Aldrich). 

To increase ADAR1-p150, cells were treated with 2000units/ml of Interferon-α (Peprotech Inc.).

### Electroporation

LB cells (4x10^6^ per well) were pelleted and resuspended in 90μl of Ingenio Electroporation Solution (Mirus) and 10μl of siRNA or plasmid. Each sample was electroporated in the Nucleofactor Device (Amaxa Biosystems) using the R-13 program. Cells were transferred into 2ml medium and cultured for a period of 24-72h; subsequently the expression of mRNA and proteins was analyzed. All experiments were repeated at least three times.

### siRNA

The siRNA used were Ambion Silencer Validated siRNAs: Negative control cat.#AM4635, ADAR1 cat.#119581 and ADAR2 cat.#119782. The optimal concentration of siRNA was 20μM for ADAR1 and 40μM for ADAR2.

### Plasmids

An ADAR1 expression vector (variant 1, pCMV6 backbone) was obtained from Origene. An empty pRc/CMV plasmid was used as control. Transfection were carried out using 1μg plasmid.

### RNA extraction and cDNA preparation

Total RNA was extracted from cells using the TRIzol reagent (Invitrogen) according to the manufacture’s protocol. cDNA was prepared from 2μg of total RNA with the High Capacity cDNA Reverse Transcription Kit (Applied Biosystems) according to the manufacture’s protocol.

### Polymerase chain reaction (PCR) amplification and sequence reactions

PCR amplifications were performed using ReddyMix PCR Master Mix (ABgene). For sequencing reactions, PCR fragments were extracted and purified from the gel using Zymoclean Gel DNA Recovery Kit (Zymo Research) according to the manufacture’s protocol. Purified DNA was fluorescently labeled using the BigDye terminator V1.1 cycle enzyme (Applied Biosystems). Following precipitation, fragments were sequenced using the ABI Prism 3100 Genetic Analyzer Biosystem.

### Analysis of A-to-I RNA editing

Inosine is similar in structure to guanine and is therefore read by the automatic DNA sequencer as “G”. A-to-I RNA editing is identified as a site in the cDNA sequence with overlapping A and G peeks. The percentage of RNA editing was measured using the Accelrys Gene software by calculating the level of guanine expression at a certain sequence position divided by the total expression of adenine and guanine at the same point. Edited sites are only those sites that exhibited more than 5% editing. 

Editing in F11R was examined in an *Alu* sequence embedded in the 3'UTR of the F11R gene. Average editing is the average percentage of editing at 12 sites (listed below). The amplified region from the *Alu* sequence is positioned at chr1: 160967771-160968006 according to the UCSC genome assembly (February 2009).

### Relative Quantification analysis

RQ-PCR was performed in order to determine the levels of mRNA expression of ADAR1-p110, ADAR1-p150, ADAR2 (ADARB1) and F11R. We used the ABL gene as a reference gene, which showed stable levels of expression in our treated and non-treated samples (data not shown). In the experiments performed with α–amanitin the 18S gene was used as a reference gene as it was not affected by the treatment. Primers were designed according to Primer-Express software guidelines (Applied Biosystems) to enable the amplification of mature mRNA only. The sequences of all the primers and probes used for RQ-PCR are listed below. All probes carried a 5'FAM and a 3'BHQ. The RQ-PCR reactions were run on ABI 7900HT genetic analyzer utilizing SDS 2.3 software (Applied Biosystems). All reactions were run as triplicates. Reactions were performed in a total volume of 20 μl containing cDNA equivalent to 100 ng of RNA from each sample. The PCR conditions consisted of 2 min at 50°C, 10 min at 95°C, 40 cycles of 15 sec at 95°C, 1 min at 60°C. Analysis of the results was performed by the SDS RQ manager 1.2 software using the ΔΔCt method. 

### Western blotting

Proteins were extracted from cells using RIPA buffer (Sigma-Aldrich) supplemented with protease inhibitor (Roche). Following separation on a SDS-PAGE, proteins were transferred to a nitrocellulose membrane using the iBlot Dry Blotting System (Invitrogen). Membranes were stained with a primary antibody over night at 4°C, washed and incubated with the appropriate secondary antibody for 45'-60' at room temperature. Specific reactive bands were detected using the SuperSignal West Pico Chemiluminescent Substrate (Thermo Scientific). Relative quantification of the proteins was performed using the Image-J software. The Antibodies used were: anti-ADAR1 (Santa Cruz sc-73408), anti-JAM-A (Santa Cruz sc-53623) and anti-Actin (Epitomics 1184-1). 

### Fractionation of cells

Fractionation was carried out using the PARIS kit (Ambion, #AM1921) according to the manufacturer's protocol. Fractionation quality was verified by carrying out relative quantification analysis of a snoRNA and by western blot analysis with an antibody against the nuclear protein emerin (data not shown).

### RNA immunoprecipitation (RIP)

LB cells were UV cross-linked (400mj/cm^2^ + 250mj/cm^2^) and fractionated using the PARIS buffer followed by sonication of the nuclear extracts. Precleared lysates were subjected to the anti-p54^nrb^ antibody or anti-mouse IgG antibody conjugated beads (anti-mouse IgG agarose beads) for 2h at 4°C in binding buffer (50mM Tris-HCl PH7.4, 150mM NaCl, 1mM MgCl_2_, 0.05% NP40 + RNAsin (200U) + 10mM DTT + 400uM RVC + 20mM EDTA). The beads were then washed 5 times with wash buffer (50mM Tris-HCl PH7.4, 150mM NaCl, 1mM MgCl_2_, 0.05% NP40) and extracted with elution buffer (100mM Tris, pH 6.8, 4% SDS, 12% β-mercaptoethanol and 20% glycerol) at room temperature for 10min. The IP material was used for RNA extraction with TRIzol. cDNA synthesis was performed as mentioned above.

### Statistical analysis

Analysis was performed using the SPSS Statistics Software for Windows (IBM). Comparative means were calculated by one-way ANOVA (Analysis of Variance) test, with significance level of p ≤ 0.01.

### Primers used in this study

Primers used for the amplification of cDNA for checking A-to-I RNA editing.

F11Redit-F: CCAAAAGGATTTAAAACCGCTGC; F11Redit-R: GAGCTGGAGT-TTTGCTCTTGTTGC; F11Rexon-F: CCCGAAGTGAAGGAGAATTCAAAC; F11Rintron-F: GTGAGGAGATTTCTAGCCCATGTT

Primers used in RQ-PCR analysis.

ABL-F: TGGAGATAACACTCTAAGCATAACTAAAGGT; ABL-R: GATGTAG-TTGGGACCCA; ADAR110-F: GTGTCCCGAGGAAGTGCAA; ADAR110-R: TGTCTGTGCTCATAGCCTTGAAA; ADAR150-F: CGGGCGCAATGAATCC; ADAR150-R: TGTGCTCATAGCCTTGAAATGG; ADAR2-F: CGCTGCGCACA-ACCAA; ADAR2-R: GTTGCCCCTTAAGCTCTCCAT; F11Rmature-F: GCCCG-AAGTGAAGGAGAATTC; F11Rmature-R: CAGATGATAGGCGGTGAGCC; 18S-F: CGGCTACCACATCCAAGGAA; 18S-R: GCTGGAATTACCGCGGCT; SnoRD57-F: GGAGGTGATGAACTGTCTGAGC; SnoRD57-R: GGATCAGGCT-CATTAAATCAGTTT

Probes used in RQ-PCR analysis

ABL-P: CCATTTTTGGTTTGGGCTTCACACCATT **;** ADAR110-P: TTCCCTCA **-**GCGGATACTACACCCATCC**;** ADAR2-P: AGACTGCGGCCGAAGCGTGG

### Editing sites in the F11R gene

Position of the editing sites used for the average editing according to UCSC genome assembly, February 2009:

1-160967833; 2-160967848; 3-160967854; 4-160967855; 5-160967856; 6-160967857; 7-160967860; 8-160967890; 9-160967891; 10-160967896; 11-160967899; 12-160967938; 

Position of the additional editing sites that appeared upon α-amanitin treatment according to UCSC genome assembly, February 2009: a-160967839; b-160967843; c-160967918; d-160967935; e-160967936; f-160967941; g-160967952;
